# Prevalence and Risk Factors of Anemia in Pregnant Women Attending Antenatal Clinic at a Medical Center in Accra, Ghana: A Cross‐Sectional Study

**DOI:** 10.1002/hsr2.72112

**Published:** 2026-03-15

**Authors:** Gifty Amewudah, Frank Twum Aboagye, Frank Quarshie, Martin Owusu Asante, Benjamin Ansah‐Agyei, Joseph Otchere, Philip Narteh Gorleku, Maurice Ofoe Gorleku, Margaret Atuahene, Abena Mantebea Ateko, Daniel Eshun

**Affiliations:** ^1^ Department of Medical Laboratory Sciences Klintaps College of Health and Allied Sciences Tema Ghana; ^2^ Biomedical and Public Health Research Unit, Water Research Institute Council for Scientific and Industrial Research Accra Ghana; ^3^ Research Directorate Klintaps College of Health and Allied Sciences Klagon‐Tema Ghana; ^4^ Department of Medical Imaging Sciences Klintaps College of Health and Allied Sciences Tema Ghana; ^5^ Department of Community Nutrition and Dietetics Klintaps College of Health and Allied Sciences Tema Ghana; ^6^ Department of Ophthalmic Dispensing Klintaps College of Health and Allied Sciences Tema Ghana

**Keywords:** anemia, antenatal care, maternal health, pregnancy, public health, risk factors

## Abstract

**Background:**

Anemia during pregnancy remains a major cause of maternal and infant morbidity in Ghana and has broader socioeconomic implications. Its persistence despite existing interventions indicates that factors such as education, occupation, and nutrition require closer evaluation. This study assessed the prevalence of anemia and its associated risk factors among pregnant women in Ghana.

**Methods:**

A hospital‐based cross‐sectional study was conducted among 152 consenting pregnant women attending antenatal care at Ark Medical Center. A structured pretested questionnaire collected demographic and clinical data. Participants from all trimesters were included. Blood samples were analyzed to determine anemia status, type, and severity. Descriptive statistics were used, and associations were evaluated using chi‐square tests and multivariable logistic regression, with *p* < 0.05 considered statistically significant.

**Results:**

Anemia was present in 55.90% of participants, of whom 63.53% had mild and 36.47% had moderate anemia. The highest prevalence occurred among women aged ≥ 36 years (65.22%) and those with secondary education (62.22%). Independent risk factors were inadequate intake of iron‐rich foods (aOR = 5.77, 95% CI: 2.46–9.28, *p* = 0.009), sickle cell disease (aOR = 6.72, 95% CI: 4.31–10.01, *p* = 0.022), and helminth infection (aOR = 5.73, 95% CI: 2.04–10.08, *p* = 0.001). Microcytic hypochromic anemia was the predominant type (47.06%), with significantly reduced mean corpuscular volume (*p* = 0.022) and mean corpuscular hemoglobin (*p* = 0.004).

**Conclusion:**

Anemia remains highly prevalent among pregnant women and is strongly associated with nutritional deficiency, sickle cell disease, and helminth infections. The dominance of microcytic hypochromic anemia supports the need for targeted nutritional support, prophylaxis, and routine screening within antenatal care to improve pregnancy outcomes.

## 1 Introduction

Anemia during pregnancy remains a significant public health challenge, affecting approximately 42% of pregnant women worldwide, with the highest prevalence reported in sub‐Saharan Africa and South Asia [1]. This condition is associated with an increased risk of maternal morbidity, adverse birth outcomes, and perinatal mortality, necessitating its prioritization in maternal healthcare interventions. The World Health Organization (WHO) defines anemia in pregnancy as a hemoglobin concentration below 11.0 g/dL [2], a threshold at which physiological adaptations to pregnancy may become insufficient to meet the increased oxygen demands. Despite global initiatives aimed at reducing anemia‐related complications through supplementation programmes, dietary interventions, and infection control, progress has been slow, particularly in resource‐limited settings [3].

The burden of anemia during pregnancy is closely linked to Sustainable Development Goal (SDG) 3, particularly Target 3.1, which aims to reduce the global maternal mortality ratio to fewer than 70 per 100,000 live births, and Target 3.2, which seeks to end preventable deaths of newborns and children under 5 years. Anemia contributes to postpartum hemorrhage, sepsis, preterm delivery, low birthweight, and perinatal death [4]. Reducing its prevalence is therefore central to progress toward these targets. In Ghana and other countries with similar socioeconomic and healthcare constraints, strengthening the detection and management of anemia in pregnancy remains essential for achieving national and global maternal and neonatal survival goals [5, 6]. By assessing the prevalence and determinants of anemia in an urban antenatal population, this study contributes evidence needed to refine maternal health interventions and to support sustained progress toward SDGs 3.1 and 3.2.

The causes of anemia in pregnancy are diverse and vary across populations. Iron deficiency is the most prevalent cause, often attributed to inadequate dietary intake, impaired absorption, or increased physiological demands [7]. In endemic regions, chronic infections such as malaria, helminthiasis, and bacterial infections contribute to anemia through mechanisms such as hemolysis, gastrointestinal blood loss, and increased hepcidin levels, which inhibit iron absorption and mobilization [8]. Additionally, genetic haemoglobinopathies, including sickle cell disease (SCD) and thalassemia, influence susceptibility to anemia through chronic hemolysis and impaired erythropoiesis [9]. Socioeconomic and dietary factors also contribute, with food insecurity and inadequate access to healthcare further exacerbating the condition. In Ghana, national health surveys estimate anemia prevalence among pregnant women to range between 28.7% and 45.0% [10, 11]. However, these statistics often fail to capture variations across different healthcare settings. Studies conducted in various regions have reported prevalence ranging from 37.0% to 78.5% [12, 13, 14], but there has been limited exploration of the combined effects of biological, nutritional, and environmental factors on anemia risk. Furthermore, few studies have assessed whether current maternal health interventions in urban healthcare settings adequately address the underlying causes of anemia.

This study examines the prevalence and associated factors of anemia among pregnant women attending antenatal care at the Ark Medical Center, Accra. By considering clinical, dietary, and demographic variables, this research aims to provide a broader assessment of the determinants of anemia in this population. Unlike previous studies that primarily focus on prevalence and in rural areas, this research evaluates the interplay of multiple contributing factors in an urban private healthcare setting, identifying unique determinants that require tailored interventions. The findings will help inform maternal health policies, support the development of targeted nutritional and infection control strategies, and enhance early detection and management protocols for anemia in pregnancy.

## 2 Materials and Methods

### 2.1 Study Design, Setting, and Population

This hospital‐based cross‐sectional study was conducted at the Ark Medical Center between February 2023 and December 2023 following the STROBE guidelines (Supporting Information S1: STROBE checklist) to determine the prevalence and risk factors associated with anemia in pregnancy. The Ark Medical Center, located in Lapaz within the Accra Metropolis of the Greater Accra Region, Ghana, is a private, well‐equipped secondary health facility. It serves as a referral center for smaller hospitals and clinics in neighboring towns and provides specialist services in antiretroviral treatment, sickle cell management, dentistry, diabetes, and hypertension. The hospital also offers antenatal and postnatal care services. The study population comprised pregnant women seeking antenatal care at the Ark Medical Center.

### 2.2 Sample Size and Sampling Technique

The sample size was estimated using the Cochrane formula for sample size estimation. The proportion was obtained based on a previous prevalence of anemia in pregnancy (11%) reported by [15] with a 95% confidence level and a 5% margin of error.

$$n=\frac{1.96^{2}\times 0.11\left(1-0.11\right)}{0.05^{2}},$$

n=150.43.

The estimated sample size was 150.43. A total of 152 pregnant women were enrolled to ensure sufficient statistical power. A post hoc power analysis confirmed that the study had robust statistical power to detect the observed prevalence of anemia (55.9%). The sample size of 152 was sufficient for identifying significant risk factors. While the initial prevalence estimate of 11% was conservative, the results highlight a higher‐than‐expected burden of anemia among pregnant women in this setting. Simple random sampling was used to recruit participants from those attending antenatal clinic sessions, with eligibility based on availability and willingness to participate.

### 2.3 Eligibility Criteria

#### 2.3.1 Inclusion Criteria

Pregnant women receiving antenatal care at the Ark Medical Center were eligible to participate, regardless of gestational age. Participants had to be at least 18 years old to provide informed consent and must have resided in Accra for at least 6 months to account for local dietary and environmental influences. All participants provided consent for blood sample collection and completion of a structured questionnaire.

#### 2.3.2 Exclusion Criteria

Women with hematological disorders (e.g., leukemia, aplastic anemia) or chronic kidney disease were excluded due to the independent effects of these conditions on hemoglobin levels. Those who had received a blood transfusion within the preceding 3 months or were on high‐dose iron therapy for unrelated conditions were also ineligible. Multiple pregnancies were excluded due to their distinct hematological profiles. To ensure consistency in healthcare access and environmental exposure, only residents of Accra attending antenatal care at the study site were included.

### 2.4 Questionnaire Administration

A structured questionnaire was administered to collect information on risk factors and relevant demographic, clinical, and dietary variables. The questionnaire comprised closed‐ended questions covering three key areas: (i) demographic characteristics and clinical history, (ii) risk factors for anemia in pregnancy, and (iii) participants' knowledge of anemia (Supporting Information S2: Structured Questionnaire).

The selection of variables included in the questionnaire was guided by major public health guidelines and relevant peer‐reviewed literature. The World Health Organization identifies inadequate dietary iron intake, increased physiological iron requirements, malaria, helminth infections, haemoglobinopathies, and previous history of anemia as principal contributors to maternal anemia in low‐ and middle‐income settings [16]. These are consistent with findings from epidemiological studies in sub‐Saharan Africa, where nutritional deficiencies, helminth infections, and SCD are common determinants [17, 18, 19, 20, 21]. Socio‐demographic variables such as age, education, employment, marital status, and parity were included because they influence maternal nutrition, health‐seeking behaviors, and overall vulnerability to anemia [22].

Dietary practices and the frequency of consuming iron‐rich foods were also assessed in line with WHO recommendations on maternal nutrition. In this study, “regular consumption of iron‐rich foods” was defined as the participant reporting consumption of at least one iron‐rich food item (such as red meat, legumes, dark green leafy vegetables, or fortified cereals) on most days of the week (≥ 4 days per week). Similarly, “regular eating of a balanced diet” was defined as consuming meals that included three major food groups (protein, carbohydrate, and vegetables/fruits) on most days of the week ( ≥ 4 days per week). Clinical history variables, including SCD, malaria, helminth infections, and prior diagnoses of anemia, were incorporated due to their direct biological effects on hemoglobin levels. SCD status was verified using participants' hospital medical records, rather than being newly diagnosed through study‐specific testing, ensuring accuracy while minimizing unnecessary repeat testing.

The questionnaire was pretested at a private maternity home using 10% of the final sample size to assess clarity, consistency, and reliability. Based on feedback, ambiguous questions were revised, response options were refined, and sections were reorganized for coherence. The final version was used for data collection. The final questionnaire was administered by trained research personnel, including laboratory scientists and research assistants, who were thoroughly familiar with the study objectives and questionnaire content. Prior to data collection, all personnel (the same who conducted the pre‐testing) underwent a 1‐day training session focused on standardized interview techniques, ethical considerations, accurate recording of responses, and clarifying participant queries without introducing bias. This ensured that data collection was consistent, reliable, and adhered to best practices for questionnaire administration in clinical research settings. Data were collected using Open Data Kit (ODK) (ODK Collect, v2023.3, University of Washington, Seattle, USA) and exported to Microsoft Excel 2019 (Microsoft Corporation, Redmond, WA, USA) for validation and cleaning.

Reliability was assessed using Cronbach's alpha [23], yielding a score of 0.813, indicating good internal consistency. Responses were scored, with correct answers assigned a value of 1 and incorrect answers assigned 0. Knowledge levels were categorized as excellent (> 80%), good (60%–80%), or poor (< 60%) following the Al‐Rubaish system of classification [24].

### 2.5 Anemia Status Assessment

Anemia status was determined using a full blood count analyzer, which measured hemoglobin concentration and red blood cell (RBC) indices, including mean corpuscular volume (MCV), mean corpuscular hemoglobin (MCH), and mean corpuscular hemoglobin concentration (MCHC). These parameters facilitated the classification of anemia types.

Venous blood sample (2 mL) was collected from each participant into ethylenediaminetetraacetic acid (EDTA) tubes following standard phlebotomy procedures. To ensure homogeneity, samples were mixed using a Stuart Scientific Blood Tube Rotator SB‐1 for 3 min before analysis. Each sample was assigned a unique identification code. Blood samples were analyzed using a three‐part hematology auto‐analyzer (Sysmex Corporation, North America), which aspirated the well‐mixed sample through an automated probe. Hemoglobin concentration, RBC count, MCV, and MCHC were recorded approximately 45 s after sample aspiration and documented in a predesigned data sheet.

Anemia was defined as hemoglobin levels below 11.0 g/dL [25]. The severity of anemia was categorized as mild (Hb: 10.0–10.9 g/dL), moderate (Hb: 7.0–9.9 g/dL), or severe (Hb < 7.0 g/dL) [25]. Microcytosis was defined as MCV < 82 fL, whereas macrocytosis was defined as MCV > 98 fL. Hypochromia was classified as MCH < 27 pg, while hyperchromia was classified as MCH > 32 pg [26].

### 2.6 Data Analysis and Study Variables

The cleaned data (Supporting Information S3) were then imported into Statistical Package for the Social Sciences (SPSS) version 27 (IBM Corp., Armonk, NY, USA) and GraphPad Prism 9.0 (GraphPad Software Inc., Boston, USA) for analysis.

Descriptive statistics were used for continuous variables, while categorical variables were summarized as percentages. The prevalence of anemia was expressed as a percentage with a 95% confidence interval (CI). Group comparisons for continuous variables were conducted using analysis of variance and chi‐square tests, where applicable.

Multivariable logistic regression was used to identify factors associated with anemia. Pregnancy trimester (first, second, third) was included in all adjusted models as a fixed‐effect covariate because of its recognized influence on hemoglobin concentration during pregnancy. Multicollinearity was evaluated using variance inflation factors, with values < 10 indicating no concerning collinearity. Crude odds ratios were first obtained from univariate logistic regression. Variables with a *p*‐value ≤ 0.20 in univariate analysis were entered into the multivariable model to estimate adjusted odds ratios (aOR) with 95% CIs. Maternal age, nutritional status, socio‐economic factors, and other relevant confounders were also included based on established clinical relevance. Model adequacy was assessed using the Hosmer–Lemeshow goodness‐of‐fit test (*χ* ^2^ = 8.773, *df* = 8, *p* = 0.362). A *p*‐value < 0.05 was considered statistically significant.

## 3 Results

### 3.1 Demographic Profile of Pregnant Women

This study included 152 pregnant women receiving antenatal care at Ark Medical Center. As summarized in Table 1, most participants (71.1%, *n* = 108) were between 26 and 35 years of age. Educational qualifications varied, with 59.2% (*n* = 90) having completed senior high school or vocational training. Most participants (80.3%, *n* = 122) were married. Regarding employment status, 54.6% (*n* = 83) were self‐employed, while 3.9% (*n* = 6) were unemployed. Gestational age distribution indicated that 47.4% (*n* = 72) of the women were in their second trimester, and 23.0% (*n* = 35) were in their third trimester. In terms of gravidity, the majority (59.9%, *n* = 91) were experiencing their first pregnancy (gravida one) at the time of the study (Table 1).

**Table 1:** Demographic characteristics of the pregnant women.

Variable	Number of participants	Percentage (%)
Age (years)		
18–25	21	13.80
26–35	108	71.10
≥ 36	23	15.10
Educational level		
JHS	12	7.90
SHS/Vocational	90	59.20
Tertiary	50	32.90
Marital status		
Single	30	19.70
Married	122	80.30
Employment status		
Civil servant	48	31.60
Self‐employed	83	54.60
Unemployed	6	3.90
Student	15	9.90
Stage of pregnancy		
First trimester	45	29.60
Second trimester	72	47.40
Third trimester	35	23.00
Gravidity		
Gravida (1) One	91	59.90
Gravida (2) Two	52	34.20
Gravida (3) Three	9	5.90

### 3.2 Nutrition and Clinical History of Pregnant Women

The nutritional assessment revealed that 92.1% (*n* = 140) of pregnant women regularly consumed iron‐rich foods, while 75.0% (*n* = 114) reported eating a balanced diet (Table 2). Clinically, 23.0% (*n* = 35) had been diagnosed with anemia before pregnancy, 17.1% (*n* = 26) had SCD, and 9.2% (*n* = 14) had a prior malaria diagnosis. Additionally, 77.0% (*n* = 117) had a history of helminth infection, whereas 23.0% (*n* = 35) did not. Regarding dietary access, 38.8% (*n* = 59) faced challenges in obtaining a balanced diet, while 61.2% (*n* = 93) had regular access (Table 2).

**Table 2:** Nutritional and clinal history of the study participants.

Variables	*n*	%
Regular consumption of iron‐rich food		
No	12	7.90
Yes	140	92.10
Prior diagnosis of anemia before pregnancy		
Yes	35	23.00
No	117	77.00
Regular eating of balanced diets		
Yes	114	75.00
No	38	25.00
Sickle cell anemia		
Sickle cell anemia	26	17.10
Normal hemoglobin	126	82.90
Previous/Current diagnosis of malaria		
Malaria positive	14	9.20
Malaria negative	138	90.80
Diagnosed with helminth infection		
Helminth infected	117	77.00
Not infected with helminth	35	23.00
Challenges with access to a balanced diet		
Challenging	59	38.80
Easy access	93	61.20

Abbreviations: %, percentage; *n*, number of pregnant women.

### 3.3 Knowledge of Pregnant Women on Anemia

**Figure 1:**
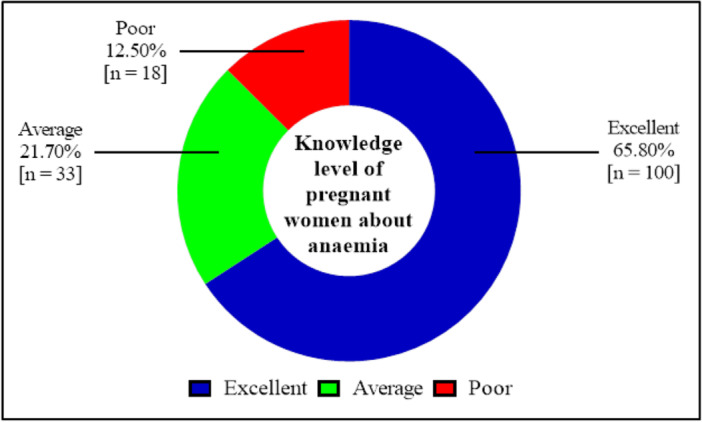
Knowledge level of the pregnant women on anemia. Blue represents pregnant women with excellent knowledge, green represents pregnant women with average knowledge of anemia, and red represents pregnant women with poor knowledge of anemia.

Knowledge of anemia among pregnant women was assessed using items on what constitutes anemia, its causes, and symptoms. Of the 152 pregnant women, 65.80% (*n* = 100) had excellent knowledge of anemia, while 12.50% (*n* = 18) had poor knowledge (Figure 1).

### 3.4 Prevalence and Severity of Anemia

**Figure 2:**
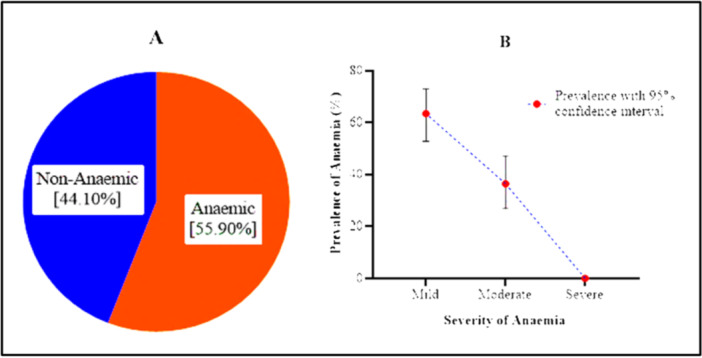
Prevalence (A) and severity (B) of anemia among the pregnant women. The figure describes the prevalence and severity of anemia among pregnant women.

Of the 152 pregnant women examined, 85 were reported to be anemic, representing a prevalence of 55.90% (95% CI: 47.97–63.58) (Figure 2A). The majority of the anemic pregnant women had mild anemia (63.58%, 95% CI: 52.88–72.98), and 36.42% had moderate anemia (95% CI: 27.02–47.12) (Figure 2B). None of the pregnant women had severe anemia. The prevalence of mild anemia was significantly different from that of moderate anemia (*χ* ^2^ = 12.467, *p* < 0.001).

### 3.5 Association of Demographic and Clinical History With Anemia Status

Although age was not significantly associated with anemia status, the highest prevalence was observed in pregnant women aged ≥ 36 years (65.22%, 95% CI: 44.68–81.20), while the lowest prevalence was recorded among those aged 18–25 years (47.62%, 95% CI: 28.22–67.79) (Table 3). Anemia was most common in the second trimester (61.11%, 95% CI: 49.51–71.55), though the association between gestational stage and anemia was not statistically significant (*p* = 0.473) (Table 3). Dietary habits showed a significant association with anemia, as women who did not consume iron‐rich foods had the highest prevalence (83.33%, 95% CI: 54.55–94.96, *p* = 0.046). Additionally, pregnant women with a prior diagnosis of anemia exhibited a notably high prevalence (88.57%, 95% CI: 73.93–95.33) (Table 3). The prevalence of anemia among pregnant women with SCD, malaria, and helminth infections were 73.08% (95% CI: 53.72–86.25), 64.29% (95% CI: 38.38–83.66), and 62.39% (95% CI: 53.33–70.65), respectively. Although no significant association was found between malaria and anemia (*p* = 0.508), there was a significant association between helminth infection and anemia (*p* = 0.003) (Table 3).

**Table 3:** Association of demographic, nutritional, and clinical characteristics of study participants with anemia.

Variables	Total number examined	Prevalence (*n* [95% CI])	*p* value
Age (years)			0.497
18–25	21	10 [47.62, 28.22–67.79]	
26–35	108	60 [55.56, 46.13–64.59]	
≥ 36	23	15 [65.22, 44.68–81.20]	
Educational level			0.098
JHS	12	04 [33.33, 13.86–61.43]	
SHS	90	56 [62.22, 51.87–71.55]	
Tertiary	50	25 [50.00, 36.60–63.40]	
Marital status			0.750
Single	30	16 [53.33, 36.04–69.84]	
Married	122	69 [61.61, 52.33–70.09]	
Employment status			0.820
Civil servant	48	28 [58.33, 44.21–71.18]	
Self‐employed	83	46 [55.42, 44.69–65.65]	
Unemployed	6	04 [66.67, 29.02–90.10]	
Student	15	07 [46.67, 24.65–70.12]	
Stage of pregnancy			0.473
First trimester	45	23 [51.11, 36.94–65.09]	
Second trimester	72	44 [61.11, 49.51–71.55]	
Third trimester	35	18 [51.43, 35.49–67.07]	
Gravidity			0.765
Gravida (1) One	91	52 [57.14, 46.86–66.83]	
Gravida (2) Two	52	29 [55.76, 42.28–68.44]	
Gravida (3) Three	9	04 [44.44, 18.71–73.76]	
Knowledge on anemia			0.925
Poor	18	10 [55.56, 33.50–75.55]	
Average	33	18 [54.55, 37.89–70.22]	
Excellent	100	57 [57.00, 47.19–66.28]	
Regular consumption of iron‐rich foods			**0.046**
No	12	10 [83.33, 54.55–94.96]	
Yes	140	75 [53.57, 45.31–61.63]	
Prior diagnosis of anemia before pregnancy			0.580
Yes	35	31 [88.57, 73.93–95.33]	
No	117	54 [46.15, 37.37–55.18]	
Regular eating of balanced diets			0.396
Yes	114	66 [57.89, 48.68–66.56]	
No	38	19 [50.00, 34.78–65.22]	
Sickle cell anemia			0.053
Sickle cell anemia	26	19 [73.08, 53.72–86.25]	
Normal hemoglobin	126	66 [52.38, 43.70–60.91]	
Prior diagnosis of malaria			0.508
Malaria positive	14	09 [64.29, 38.38–83.66]	
Malaria negative	138	76 [55.07, 46.73–63.13]	
Diagnosed with helminth infection			**0.003**
Helminth infected	117	73 [62.39, 53.33–70.65]	
Not infected with helminth	35	12 [34.29, 20.82–50.97]	
Challenges with access to a balanced diet		0.313	
Challenging	59	36 [61.02, 48.21–72.43]	
Easy access	93	49 [52.69, 42.61–62.54]	

*Note:* Values reported are the number of positives (*n*), prevalence (%), and 95% confidence interval. The bold values are *p*‐values that are statistically significant at *p* < 0.05.

### 3.6 Distribution of Red Blood Cells, Hemoglobin, and Red Blood Cell Indices

**Figure 3:**
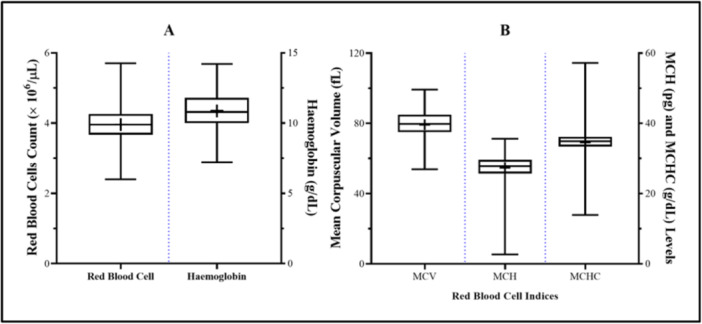
Distribution of RBC, Hb (A), and RBC indices (B) among the pregnant women. The figure shows the mean and standard deviation of the Hemoglobin (Hb) level and the red blood cells (RBC) and RBC indices among the pregnant women.

Figure 3 presents the distribution of RBC count, hemoglobin levels, and RBC indices among pregnant women. The mean RBC count and hemoglobin levels were 3.96 ± 0.55 × 10^6^/µL and 10.87 ± 1.34 g/dL, respectively (Figure 3A). The mean MCV, MCH, and MCHC were 79.28 ± 7.93 fL, 27.39 ± 3.94 pg, and 34.56 ± 4.23 g/dL, respectively (Figure 3B).

**Figure 4:**
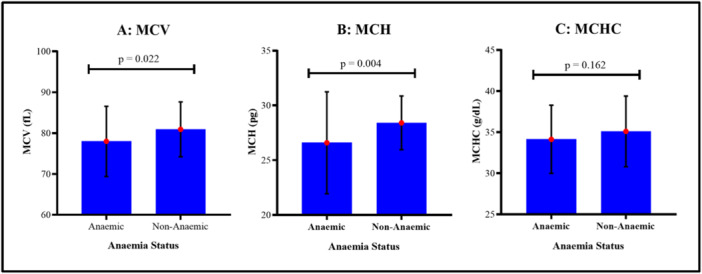
Comparison of the RBC indices [(A) MCV; (B) MCH; (C) MCHC] between anemic and healthy pregnant women.

Figure 4 compares RBC indices between anemic and non‐anemic pregnant women. Non‐anemic women had a significantly higher mean MCV (80.93 ± 6.71 fL) than anemic women (77.98 ± 8.59 fL, *p* = 0.022) (Figure 4A). Similarly, the mean MCH was significantly higher in non‐anemic women (28.41 ± 2.45 pg) compared to anemic women (26.59 ± 4.65 pg, *p* = 0.004) (Figure 4B). Although the mean MCHC was higher in non‐anemic women (35.10 ± 4.30 g/dL) than in anemic women (34.13 ± 4.15 g/dL), the difference was not statistically significant (*p* = 0.162) (Figure 4C).

### 3.7 Prevalence of the Types of Anemia Among Pregnant Women

**Figure 5:**
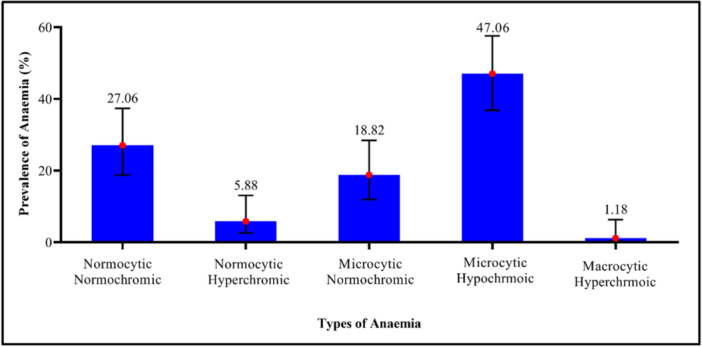
Morphological classification of anemia among pregnant women.

Among the 81 anemic cases, 47.06% (95% CI: 36.78–57.59) were classified as microcytic hypochromic anemia, while 27.06% (95% CI: 18.76–37.37) had normocytic normochromic anemia. Also, 18.82% (95% CI: 11.96–28.45) were classified as microcytic normochromic anemia, 5.88% (95% CI: 2.60–13.05) had normocytic hyperchromic anemia, and 1.18% (95% CI: 0.28–6.31) were diagnosed with macrocytic hyperchromic anemia (Figure 5).

### 3.8 Risk Factors and Predictors Associated With Anemia in Pregnant Women

A multivariate regression model identified dietary intake and clinical conditions as key predictors of anemia. Pregnant women who did not regularly consume iron‐rich foods were 5.774 times more likely to develop anemia (95% CI: 2.464–9.284, *p* = 0.009). The presence of SCD (6.720 times higher risk, *p* = 0.022) and helminth infections (5.733 times higher risk, *p* = 0.001) were also significantly associated with anemia. Demographic factors such as age, education, marital status, and employment did not show statistically significant associations with anemia. When trimester was included as a fixed‐effect covariate in the multivariable model, neither first‐ nor second‐trimester status was significantly associated with anemia compared with third‐trimester women. aORs were: first trimester aOR = 1.276 (95% CI: 0.420–3.872; *p* = 0.667) and second trimester aOR = 1.188 (95% CI: 0.452–3.125; *p* = 0.726). These findings persisted after adjustment for age, educational level, parity, dietary variables, SCD, and helminth infection. Pregnant women with poor knowledge of anemia had an increased but non‐significant risk of developing anemia (aOR = 1.485, 95% CI: 0.369–5.976) as compared to those with average knowledge (aOR = 1.038, 95% CI: 0.370–2.909) (Table 4).

**Table 4:** Risk factors and predictors of anemia among pregnant women.

Variables	Crude			Adjusted		
	Odds ratio	95% CI	*p* value	Odds ratio	95% CI	*p* value
Age (years)						
18–25	0.717	0.324–1.586	0.411	0.541	0.109–2.675	0.451
26–35	1.630	1.045–2.541	**0.018**	0.584	0.169–2.026	0.397
≥ 36	1			1		
Educational level						
JHS	0.394	0.124–1.253	**0.019**	0.478	0.053–4.304	0.510
SHS	1.298	0.980–1.720	0.061	3.486	0.935–12.993	0.063
Tertiary	1			1		
Marital status						
Single	0.901	0.474–1.712	0.750	0.587	0.182–1.889	0.372
Married	1			1		
Employment status						
Civil servant	1.104	0.685–1.777	0.684	0.866	0.224–3.358	0.836
Self‐employed	0.980	0.732–1.311	0.892	0.294	0.048–1.821	0.188
Unemployed	1.576	0.298–8.349	0.592	0.607	0.039–9.556	0.723
Student	1			1		
Stage of pregnancy						
First trimester	0.824	0.505–1.344	0.439	1.276	0.420–3.872	0.667
Second trimester	1.239	0.874–1.756	0.222	1.188	0.452–3.125	0.726
Third trimester	1			1		
Gravidity						
Gravida (1) One	1.051	0.807–1.369	0.711	2.464	0.397–5.293	0.333
Gravida (2) Two	0.994	0.638–1.549	0.978	2.070	0.349–7.277	0.423
Gravida (3) Three	1			1		
Knowledge on anemia						
Poor	0.985	0.412–2.358	0.974	1.485	0.369–5.976	0.578
Average	0.946	0.516–1.734	0.857	1.038	0.370–2.909	0.943
Excellent	1			1		
Regular consumption of iron‐rich food						
No	3.941	0.864–17.383	**0.046**	5.774	2.464–9.284	**0.009**
Yes	1			1		
Prior diagnosis of anemia before pregnancy						
Yes	1.182	0.652–2.145	0.580	0.472	0.116–1.920	0.294
No	1			1		
Regular eating of balanced diets						
Yes	1.084	0.897–1.309	0.396	1.865	0.641–5.431	0.253
No	1			1		
Sickle cell anemia						
Sickle cell anemia	2.139	0.956–4.787	0.053	6.270	4.310–10.009	**0.022**
Normal hemoglobin	1			1		
Prior diagnosis of malaria						
Malaria positive	1.419	0.499–4.035	0.508	1.866	0.436–7.991	0.400
Malaria negative	1			1		
Diagnosed with helminth infection						
Helminth infected	1.308	1.078–1.587	**0.003**	5.733	2.044–10.084	**0.001**
Not infected with helminth	1			1		
Challenges with access to a balanced diet						
Challenging	1.234	0.816–1.866	0.313	1.588	0.677–3.722	0.288
Easy access	1			1		

*Note:* 95% CI: 95% confidence interval. The bold values are *p*‐values that are statistically significant at *p* < 0.05.

## 4 Discussion

This study examined the prevalence and associated factors of anemia among pregnant women attending antenatal care at Ark Medical Center. The findings indicate a prevalence of 55.90% (95% CI: 47.97–63.58), which reflects the ongoing burden of maternal anemia. The prevalence of anemia in this study (55.9%) is similar to findings from rural districts like Adaklu (56.8%) [14], but much higher than reports from semi‐urban areas like Berekum (11.4%) [20]. The variation likely reflects differences in healthcare access, dietary patterns, and intervention coverage between urban, semi‐urban, and rural populations. Comparisons with data from Malaysia (57.4%) [27] and China (43.59%) [28] demonstrate that maternal anemia remains a widespread public health issue that requires tailored intervention strategies.

The findings suggest that maternal anemia is not solely influenced by dietary iron intake but may also be affected by inflammation, gut microbiome composition, and psychosocial stress. The higher prevalence of anemia among women aged ≥ 36 years (65.22%) compared to younger women (47.62%) may be associated with age‐related changes in iron metabolism, as observed in studies from China [28] and Tanzania [29]. Incorporating biomarker‐based screening, including inflammatory markers, could improve early detection and facilitate more targeted prevention efforts.

Academic qualification did not show a statistically significant association with anemia (*p* = 0.098), though women with secondary education (SHS/Vocational) had the highest prevalence (62.22%), whereas those with Junior High School (JHS) education had the lowest (33.33%). Socio‐economic factors such as employment opportunities, health literacy, and access to healthcare services may partly explain this trend. Women with secondary education may experience greater financial constraints than those with tertiary education, limiting their ability to maintain an adequate diet and access healthcare services [30]. This finding is consistent with studies in Bangladesh [31], where formal education alone did not correlate with reduced anemia prevalence. Addressing economic barriers, strengthening maternal health education, and promoting affordable nutritional supplementation could be more effective in reducing anemia rates.

Marital and employment status were not significantly associated with anemia (*p* = 0.750 and *p* = 0.820, respectively). However, the highest prevalence was observed among married women (61.61%) and unemployed women (66.67%). These patterns may indicate the role of household economic stability and food security in determining dietary quality and access to maternal healthcare. Similar observations have been reported in Ghana [32] and Tanzania [29]. Financial instability and chronic stress may contribute to alterations in iron metabolism [12], supporting the need for policies aimed at improving economic security and healthcare access for pregnant women.

Regular consumption of iron‐rich foods was associated with a lower risk of anemia (*p* = 0.046). Women with a prior diagnosis of anemia (88.57%), SCD (73.08%), malaria (64.29%), and helminth infections (62.39%) exhibited a higher prevalence of anemia, with helminth infections showing a significant association (*p* = 0.003). Although routine deworming programmes exist, they may not fully address the role of inflammation and nutrient absorption in anemia development. Evaluating the efficacy of current deworming strategies and considering additional interventions targeting inflammatory pathways could improve outcomes.

SCD was strongly associated with anemia (73.08%). This condition contributes to anemia through mechanisms beyond iron deficiency, including chronic hemolysis, vaso‐occlusion, and impaired erythropoiesis [33]. The elevated hepcidin levels in individuals with SCD further disrupt iron homeostasis by inhibiting absorption and recycling [34, 35]. Standard iron supplementation may not be appropriate in this population, necessitating alternative approaches such as erythropoiesis‐stimulating agents, folic acid supplementation, and anti‐inflammatory therapies.

Malaria remains a recognized contributor to anemia, with prevalence rates of 63.5% in Akatsi South [32] and 74% in Ada West [36]. However, research from Eastern Sudan [37] suggests that in low‐transmission areas, malaria may play a lesser role in anemia development. In this study, the association between malaria and anemia was not statistically significant (*p* = 0.508), but the biological mechanisms through which Plasmodium infection leads to anemia, including hemolysis, bone marrow suppression, and cytokine‐induced iron sequestration, remain relevant [38]. The inconsistency in statistical significance may reflect variations in malaria transmission intensity, immunity, and co‐existing nutritional deficiencies. Given that iron supplementation can increase iron availability for the parasite [39], a personalized approach to iron therapy, informed by biomarkers of iron status and infection risk, may be beneficial.

Helminth infections, particularly those caused by Ascaris lumbricoides and hookworms, remain a contributing factor to maternal anemia [40, 41]. These infections impair nutrient absorption and elevate systemic inflammation. Research on the gut‐liver axis has shown that hepatic iron storage and inflammatory responses are interlinked [42, 43], suggesting that addressing gut health may improve iron metabolism.

Despite ongoing maternal health interventions, anemia remains highly prevalent among pregnant women in Ghana. This suggests potential gaps in the implementation and effectiveness of current strategies. National guidelines recommend iron and folic acid supplementation, routine deworming, and malaria prophylaxis for pregnant women. However, adherence to supplementation regimens is often poor due to side effects, misinformation, and limited access to antenatal care services, particularly in resource‐constrained settings. Additionally, while deworming and malaria prevention efforts exist, their coverage and integration into routine antenatal visits may not be optimal, leaving gaps in infection control. Another key limitation is the predominant focus on iron supplementation, while other contributing factors, such as chronic inflammation, micronutrient deficiencies (e.g., vitamin B12, folate), and genetic haemoglobinopathies, are not systematically addressed. Strengthening health education, ensuring consistent availability of nutritional supplements, and adopting a more holistic, multi‐pronged approach to anemia management are essential for improving maternal health outcomes.

The findings of this study indicate that maternal anemia is influenced by a combination of dietary, infectious, inflammatory, and socio‐economic factors. Conventional screening methods that rely solely on hemoglobin levels may not provide a complete assessment of anemia risk [44, 45]. The integration of additional biomarkers, such as ferritin, C‐reactive protein, and interleukin‐6, may enhance risk stratification and guide more targeted interventions. Implementing these interventions can directly support the achievement of SDGs 3.1 and 3.2 by reducing maternal mortality and preventing adverse neonatal outcomes in Ghana and other countries with similar healthcare and socio‐economic contexts. Addressing anemia through a multidisciplinary approach will contribute to improved maternal and neonatal health outcomes.

### 4.1 Limitations and Strengths of the Study

This study has several limitations. Its cross‐sectional design does not allow for the determination of causality between anemia and its associated factors. Additionally, the reliance on self‐reported dietary intake introduces the possibility of recall bias, which could affect the accuracy of dietary assessments. Some aORs had wide CIs, suggesting potential overfitting or sample size limitations. Increasing the sample size and applying penalized regression techniques in future studies could improve the robustness of statistical estimates. Although all participants were screened for HIV as part of routine antenatal care, none tested positive; consequently, the study could not evaluate the contribution of HIV infection to anemia in pregnancy, which limits the assessment of this important risk factor. Furthermore, the study did not assess folic acid and vitamin B12 status, which are known contributors to anemia in pregnancy. The absence of biochemical measurements for these micronutrients may have led to an underestimation of nutritional risk factors. Future studies should include biomarker‐based nutritional assessments to provide a more comprehensive evaluation. Anemia classification relied solely on instrument‐based measurements of hemoglobin and red blood cell indices, which, while accurate and reproducible, may not fully capture subtle morphological variations that could help distinguish specific types of anemia. Future studies could incorporate morphological examinations to provide a more comprehensive characterization of anemia in pregnancy.

Despite these limitations, the study has several strengths. The use of laboratory‐confirmed hemoglobin measurements reduces the likelihood of misclassification compared to self‐reported anemia diagnoses. By incorporating biochemical, socio‐economic, and dietary variables, the analysis provides a comprehensive assessment of factors associated with anemia beyond hemoglobin levels alone. The application of multivariate logistic regression helped control for potential confounders, strengthening the validity of observed associations. Although some odds ratios had wide CIs, the study effectively identifies key risk factors relevant to maternal health in Ghana. Future research should employ prospective cohort designs, biomarker‐based nutritional assessments, and a broader range of healthcare settings to improve generalizability.

## 5 Conclusion

Anemia remains a significant public health concern among pregnant women attending antenatal care at Ark Medical Center. This study identified nutritional deficiencies, SCD, and helminth infections as key contributing factors. Inadequate intake of iron‐rich foods, SCD, and helminth infections were significantly associated with anemia. The predominance of microcytic hypochromic anemia suggests that iron deficiency is a primary concern, warranting improved nutritional interventions. Given that the highest prevalence was observed in the second trimester and among primigravidae, targeted interventions should prioritize early antenatal visits to facilitate timely screening and treatment. A comprehensive public health strategy incorporating nutritional supplementation, malaria prophylaxis, deworming, and routine screening is necessary to reduce the burden of anemia in pregnancy. Future research should examine the socio‐economic and behavioral determinants of anemia to inform policies that enhance maternal healthcare services. Addressing anemia through a multidisciplinary approach will contribute to improved maternal and neonatal health outcomes.

## Author Contributions

**Gifty Amewudah:** data curation, investigation, writing – original draft, writing – review and editing. **Frank Twum Aboagye:** conceptualization, data curation, formal analysis, investigation, methodology, validation/visualization, writing – original draft, writing – review and editing. **Frank Quarshie:** formal analysis, methodology, project administration, validation/visualization, writing – review and editing. **Martin Owusu Asante:** conceptualization, formal analysis, methodology, project administration, resources, validation/visualization, writing – original draft, writing – review and editing. **Benjamin Ansah‐Agyei:** investigation, methodology, resources, writing – review and editing. **Joseph Otchere:** conceptualization, resources, writing – review and editing. **Philip Narteh Gorleku:** resources, writing – review and editing. **Maurice Ofoe Gorleku:** methodology, writing – review and editing. **Margaret Atuahene:** methodology, project administration, writing – review and editing. **Abena Mantebea Ateko:** methodology, resources, writing – review and editing. **Daniel Eshun:** methodology, resources, writing – review and editing.

## Ethics Statement

The study protocol (Protocol No.: KCOHASERC/EC/2023/012) was approved by the Ethical Review Committee of the Klintaps College of Health and Allied Sciences. The study was conducted in accordance with the Declaration of Helsinki.

## Consent

Informed and written consent was obtained from all study participants before inclusion in the study. Participants were assured of the strict confidentiality and safety of any information they provided for the study.

## Conflicts of Interest

The authors declare no conflicts of interest.

## Transparency Statement

The lead authors, Frank Twum Aboagye and Frank Quarshie, affirm that this manuscript is an honest, accurate, and transparent account of the study being reported; that no important aspects of the study have been omitted; and that any discrepancies from the study as planned (and, if relevant, registered) have been explained.

## Supporting information


**Supporting File 1:** STROBE Checklist.

**Supporting File 2:** Structured Questionnaire.

**Supporting File 3:** Research Data.

## Data Availability

The data that supports the findings of this study are available in the Supporting Material of this article (Supporting Information S3).
