# Supplementary material for: Prevalence and Risk Factors of Anemia in Pregnant Women Attending Antenatal Clinic at a Medical Center in Accra, Ghana: A Cross‐Sectional Study
Source: Health Sci Rep. 2026 Mar 15;9(3):e72112. doi: 10.1002/hsr2.72112 (PMC13097454; doi:10.1002/hsr2.72112)
Supplement: Supplementary file 2 — Supporting File 2: Structured Questionnaire. [file HSR2-9-e72112-s003.docx]

**DATA COLLECTION INSTRUMENT**

Prevalence and Risk Factors of Anaemia in Pregnant Women Attending Antenatal at the Ark Medical Centre. Please answer the following questions by marking the appropriate answer(s) with an √.

This questionnaire is strictly for research purposes only.

**SECTION A: Demographic Information of Respondents (Please tick where appropriate)**

1. Please indicate your marital status
2. Single [b] Married [c] Divorced [d] Widowed
3. Please indicate your age category in years

[a] Below 20 [b] 21 – 25 [c] 26 – 30 [d] 36 and above

1. Please indicate your highest level of education

[a] JHS [b] SHS [c] Diploma [d] Degree [e] Postgraduate

[f] Others (please specify………………………………………………………..

1. Please indicate your type of employment

[a] Self-employed [b] Civil Servant [c] Private Employee [d] Unemployed

[f] Student

1. Please indicate your stage of pregnancy

[a] First Trimester [b] Second Trimester [c] Third Trimester

**SECTION B: Knowledge about anaemia (Please tick where appropriate)**

1. What is anaemia?

[a] Poor Nutrition [b] Low Haemoglobin [c] Iron Deficiency [d] Loss of Appetite

1. What is/are the signs and symptoms of anaemia? (*Tick as many as apply*)

[a] Shortness of Breath [b] Exceptional Fatigue [c] General Body Weakness

[d] Loss of Appetite [e] Dizziness or Fainting [f] I don’t know

1. Is anaemia normal in pregnancy?

[a] Yes [b] No

1. Is it possible to prevent anaemia?

[a] Yes [b] No

1. Poor nutrition causes anaemia.

[a] Yes [b] No

1. Malaria causes anaemia.

[a] Yes [b] No

1. Bleeding during pregnancy causes anaemia.

[a] Yes [b] No

1. Taking prescribed medication causes anaemia.

[a] Yes [b] No

1. Drinking caffeine or tea causes anaemia.

[a] Yes [b] No

1. Pica causes anaemia

[a] Yes [b] No

**SECTION C: Risk factors to anaemia (Please tick where appropriate)**

1. Have you been diagnosed with anaemia before this pregnancy?

[a] Yes [b] No

1. Do you consume iron-rich foods such as red meat, poultry, fish, beans, and fortified cereals regularly during pregnancy?

[a] Yes [b] No

1. Have you been diagnosed with any chronic medical conditions such as chronic kidney disease or autoimmune disorders before pregnancy?

[a] Yes [b] No

1. Do you follow a balanced diet that includes a variety of fruits, vegetables, and whole grains during pregnancy?

[a] Yes [b] No

1. Have you or your partner been diagnosed with sickle cell disease or thalassemia?

[a] Yes [b] No

1. Have you been diagnosed with any infections such as malaria during pregnancy?

[a] Yes [b] No

1. Have you been diagnosed with any infections such as helminth infestations during pregnancy?

[a] Yes [b] No

1. Do you face any challenges accessing nutritious foods, prenatal care, or iron supplementation due to socioeconomic factors?

[a] Yes [b] No

Thank you for participating in this study
